# Whey Peptides Stimulate Differentiation and Lipid Metabolism in Adipocytes and Ameliorate Lipotoxicity-Induced Insulin Resistance in Muscle Cells

**DOI:** 10.3390/nu12020425

**Published:** 2020-02-06

**Authors:** Kenneth D’Souza, Angella Mercer, Hannah Mawhinney, Thomas Pulinilkunnil, Chibuike C. Udenigwe, Petra C. Kienesberger

**Affiliations:** 1 Department of Biochemistry and Molecular Biology, Faculty of Medicine, Dalhousie University, Dalhousie Medicine, Saint John, NB E2L 4L5 Canadaan219892@dal.ca (A.M.); tpulinil@dal.ca (T.P.); 2 Department of Plant, Food, and Environmental Sciences, Faculty of Agriculture, Dalhousie University, Truro, NS B2N 5E3, Canada; hmawhinn@stfx.ca; 3 School of Nutrition Sciences, Faculty of Health Sciences, University of Ottawa, Ottawa, ON K1H 8M5, Canada

**Keywords:** whey peptides, adipocytes, myocytes, differentiation, metabolism, lipolysis, lipotoxicity, mitochondria, insulin resistance, PPAR

## Abstract

Deregulation of lipid metabolism and insulin function in muscle and adipose tissue are hallmarks of systemic insulin resistance, which can progress to type 2 diabetes. While previous studies suggested that milk proteins influence systemic glucose homeostasis and insulin function, it remains unclear whether bioactive peptides generated from whey alter lipid metabolism and its accumulation in muscle and adipose tissue. Therefore, we incubated murine 3T3-L1 preadipocytes and C2C12 myotubes with a whey peptide mixture produced through pepsin-pancreatin digestion, mimicking peptides generated in the gut from whey protein hydrolysis, and examined its effect on indicators of lipid metabolism and insulin sensitivity. Whey peptides, particularly those derived from bovine serum albumin (BSA), promoted 3T3-L1 adipocyte differentiation and triacylglycerol (TG) accumulation in accordance with peroxisome proliferator-activated receptor γ (PPARγ) upregulation. Whey/BSA peptides also increased lipolysis and mitochondrial fat oxidation in adipocytes, which was associated with the upregulation of peroxisome proliferator-activated receptor δ (PPARδ). In C2C12 myotubes, whey but not BSA peptides ameliorated palmitate-induced insulin resistance, which was associated with reduced inflammation and diacylglycerol accumulation, and increased sequestration of fatty acids in the TG pool. Taken together, our study suggests that whey peptides generated via pepsin-pancreatin digestion profoundly alter lipid metabolism and accumulation in adipocytes and skeletal myotubes.

## 1. Introduction

Increased consumption of calorically dense foods coupled with a sedentary lifestyle can lead to obesity, a chronic metabolic disorder characterized by the accumulation of excessive adipose tissue. Obesity is associated with many comorbidities, including cancer, cardiovascular disease, and insulin resistance, which can progress to type 2 diabetes. Systemic insulin resistance is preceded by the development of insulin resistance in key insulin target tissues, such as skeletal muscle and white adipose tissue (WAT) [1].

The skeletal muscle acts as a primary site of insulin-stimulated glucose transport, accounting for 25–30% of postprandial glucose disposal [2]. During insulin resistance, muscle glucose uptake is significantly reduced; chronically, this can promote systemic impairment of glucose homeostasis and hyperglycemia [1]. WAT primarily serves as a postprandial nutrient sink, sequestering excess nutrients in the form of triacylglycerols (TGs) [3]. Insulin promotes TG synthesis and suppresses enzymes involved in TG hydrolysis, thereby lowering fatty acid release from adipocytes; however, these functions are impaired in insulin-resistant adipose tissue [4]. The inability of WAT to effectively expand in the presence of excess nutrients promotes ectopic lipid accumulation in tissues such as skeletal muscle [5]. WAT can also influence systemic insulin sensitivity through the secretion of peptide and lipid hormones known as adipokines and lipokines, respectively [6,7]. The adipokine profile is drastically altered in insulin resistance, with increased secretion of inflammatory adipokines, such as tumor necrosis factor α (TNFα), and decreased secretion of insulin-sensitizing adipokines, such as adiponectin [8]. The mechanisms implicated in insulin resistance in skeletal muscle and WAT are multifactorial and involve increased inflammation [9,10], oxidative stress [11], fibrosis [12], endoplasmic reticulum (ER) stress [13], and accumulation of toxic signaling lipids, including diacylglycerols (DGs) and ceramides [14,15].

Considerable work has focused on utilizing functional food agents, such as bioactive peptides, for the prevention and treatment of insulin resistance. Bioactive peptides are short amino acid sequences derived from a variety of food sources, including milk, egg, soy, nuts, and peas [16]. Bioactive peptides can be generated in the gastrointestinal system through the digestion of dietary proteins with enzymes such as pepsin and pancreatic proteases, or exogenously through food processing such as enzymatic treatments and fermentation [16,17]. Prior studies showed that bioactive peptides could influence adipose tissue and skeletal muscle metabolism and potentially have insulin-sensitizing effects. For example, tripeptides isolated from the bovine milk protein casein or egg white hydrolysates promote adipocyte differentiation and expansion, along with reducing inflammation [18,19,20]. Overall, peptides derived from bovine casein, egg white protein, and soy protein appear to have an insulin-sensitizing effect in rodent models and cultured cells, although the underlying molecular mechanisms remain unclear [21,22].

Whey protein, the second most abundant milk protein representing 18–20% of milk nitrogen content [23], has also gained interest as a source of bioactive peptides. Whey protein mainly consists of β-lactoglobulin (50%), α-lactalbumin (20%), and serum albumin (10%) [23], all of which can serve as sources of bioactive peptides. Whey peptide supplementation was shown to reduce fat mass in senescent mice [24]. Hydrolysate from bovine α-lactalbumin ameliorated high fat diet-induced body weight gain and improved systemic glucose homeostasis in mice [25]. A β-lactoglobulin-derived peptide reduced hepatic TG and cholesterol accumulation in zebrafish [26]. In skeletal muscle, the consumption of whey protein or peptides resulted in increased glycogen levels in mice and rats and adenosine monophosphate-activated protein kinase (AMPK) phosphorylation in mice [24,27,28]. Exposure to whey-derived dipeptides also stimulated non-insulin dependent glucose uptake in L6 myotubes and isolated rat muscle [29]. While these studies suggest that whey peptides influence baseline glucose homeostasis, it remains unclear whether the peptides affect insulin sensitivity and lipid metabolism in muscle and adipose tissues.

Therefore, we set out to understand whether whey peptides influence insulin function and lipid homeostasis in adipocytes and skeletal myotubes. We employed whey peptides as a mixture to mimic bioactive peptides generated in the gut from whey protein as “functional food” and examined their effect on measures of lipid accumulation and mitochondrial metabolism, insulin sensitivity, and inflammation. Our study shows that whey peptides, particularly those derived from the bovine serum albumin (BSA) component of whey, promoted 3T3-L1 adipocyte differentiation and lipid accumulation. Whey/BSA peptides also stimulated lipolysis and mitochondrial fat oxidation in adipocytes. In C2C12 myotubes, whey but not BSA peptides ameliorated palmitate-induced insulin resistance, which was associated with reduced inflammation and DG levels.

## 2. Materials and Methods

### 2.1. Chemicals and Reagents

Unless otherwise stated, chemicals and reagents were obtained from Sigma.

### 2.2. Preparation of Protein and Peptide Mixtures

BSA (fatty acid-free) and whey protein powder (Bulk Barn, Ottawa, Ontario, Canada) were dispersed in distilled water at 5% (w/v), stirred, heated to 37 °C, and adjusted to pH 2.0 using 2 M HCl. Thereafter, pepsin (from porcine stomach mucosa) was added to the protein suspensions at an enzyme-substrate ratio (E/S) of 1:100 (w/w) followed by stirring for 2 h. After peptic digestion, the hydrolysate solutions were adjusted to pH 7.5 using 2 M NaOH, and pancreatin (from the porcine pancreas) was added at an E/S of 1:100 (w/w) to simulate intestinal digestion. After 3 h of stirring, hydrolysis was terminated by heating the reaction vessels at 90 °C for 15 min to inactivate the digestive enzymes. The hydrolysates were then cooled to room temperature, frozen at -80 °C, and freeze-dried to obtain the hydrolysate powders. The unhydrolyzed BSA and whey protein isolates are referred to as BPI and WPI, respectively. Peptide-containing BSA and whey protein hydrolysates are referred to as BPH and WPH, respectively. Shotgun-based proteomics was used to identify peptides in WPH. Liquid chromatography-tandem mass spectrometry was conducted at the John L. Holmes Mass Spectrometry Facility, the University of Ottawa, using a Thermo Scientific Orbitrap Fusion-Electron transfer dissociation Orbitrap mass spectrometer (Thermo Fisher Scientific, San Jose, CA, USA). LC-MS/MS data were processed with the MaxQuant version 1.6.10.43 software (Max Planck Institute of Biochemistry, Planegg, Germany) utilizing the Andromeda peptide search engine. The spectral data analysis was performed using the approach reported by Tyanova et al. [30].

### 2.3. Cell Culture

3T3-L1 preadipocytes (ATCC) were grown and differentiated to mature adipocytes, as described [31]. Briefly, 2.0 × 10^5^ cells were seeded in six-well dishes and maintained in Dulbecco’s modified Eagle medium (DMEM) containing 25 mM glucose (DMEM-HG) (SH3024301, Fisher, Hampton, NH, USA), supplemented with 10% fetal bovine serum (FBS) (Avantor, Radnor Township, PA, USA). Two days post-confluency (day 0 of differentiation), cells were differentiated in DMEM-HG + 10% FBS, 10 mg/mL insulin (from bovine pancreas, Sigma, Saint Louis, MO, USA), 0.4 mg/mL dexamethasone, and 0.5 mM 3-isobutyl-1-methylxanthine. On day 2 of differentiation, the media was changed to DMEM-HG + 10% FBS and 10 mg/mL insulin. After 2 days (day 4 of differentiation), DMEM-HG + 10% FBS and 0.5 mg/mL insulin was added. From day 6 of differentiation, cells were maintained in DMEM-HG + 10% FBS until collection on day 8. To examine the effect of whey protein and peptides on adipocyte differentiation, media was supplemented with 2.5 mg/mL of BPI, WPI, BPH, or WPH from day 0 to day 8 of differentiation.

C2C12 myoblasts (ATCC) were grown and differentiated to myotubes as described [32]. Briefly, 5.0 × 10^5^ cells were seeded in 60 mm plates and maintained in DMEM-HG supplemented with 10% FBS. After 24 h, myoblasts were differentiated in DMEM-HG + 0.2% FBS. To induce insulin resistance, myotubes were incubated in DMEM (11966025, Thermo Fisher Scientific, Waltham, MA, USA) containing 5 mM glucose, 2% (w/v) fatty acid-free (FAF) BSA, and 0.4 mM sodium palmitate for 18 h, as described [32]. Control myotubes were cultured in the absence of palmitate. Media was also supplemented with 2.5 mg/mL of BPI, BPH, WPI, or WPH. To examine insulin signaling, cells were incubated with 100 nM insulin or an equivalent volume of phosphate-buffered saline (PBS) for 15 min. Harvested cells were flash-frozen in liquid nitrogen and stored at -80 °C until further use.

### 2.4. Gene Expression Analysis

RNA isolation, reverse transcription, and real-time quantitative PCR were performed as previously described [31,33]. The primer sequences used are summarized in Table 1.

### 2.5. Immunoblotting Analysis

3T3-L1 and C2C12 cells were homogenized in lysis buffer (20 mM Tris-HCl pH 7.5, 5 mM EDTA, 10 mM Na_4_P_2_O_7_, 100 mM sodium fluoride, 1% (v/v) NP-40) containing 2 mM sodium orthovanadate, 2 mM protease inhibitor cocktail (P8340, Sigma, Saint Louis, MO, USA), and 100 µg/mL phosphatase inhibitor cocktail (524628, Calbiochem, Saint Louis, MO, USA) by sonication. Protein content in the cell lysates was determined using a bicinchoninic acid (BCA) protein assay kit (Thermo Fisher Scientific, Waltham, MA, USA). Equal (24 µg) amounts of lysate protein were subjected to SDS-PAGE, and proteins were transferred onto a nitrocellulose membrane. Proteins were visualized using a reversible protein stain (Memcode, Thermo Fisher Scientific, Waltham, MA, USA). Membranes were incubated with the following primary antibodies: anti-PPARγ (2435, Cell Signaling, Danvers, MA, USA), anti-C/EBPα (8178, Cell Signaling), anti-adiponectin (NBP2-22450, Novus Biologicals, Centner, CO, USA), anti-pHSL ^S660^ (4126, Cell Signaling), anti-HSL (4107, Cell Signaling), anti-ATGL (2138, Cell Signaling), anti-Perilipin-1 (9349, Cell Signaling), anti-pAKT ^S473^ (9271, Cell Signaling), anti-AKT (05-591, Millipore, Burlington, MA, USA), anti-Glut4 (07-140, Millipore), anti-CHOP (sc-7351, Santa Cruz Biotechnology, Dallas, TX, USA), anti-pJNK ^T183/Y185^ (4688, Cell Signaling), and anti-JNK (9252, Cell Signaling). Immunoblots were developed using the Western Lightning Plus-ECL enhanced chemiluminescence substrate (Perkin Elmer, Waltham, MA, USA). Densitometric analysis was performed using Image Lab software (Bio-Rad, Hercules, CA, USA).

### 2.6. Lipid Analysis

For targeted lipidomic analysis, 5.0 × 10^5^ C2C12 cells and 2.0 × 10^5^ 3T3-L1 cells were spiked with 10 μL of internal standard solution (containing 10 μM ISTD, DG 14:0/14:0, 50 μM TG 15:0/15:0/15:0 and 10 μM TG 17:0/17:0/17:0) (Avanti Polar Lipids, Alabaster, AL, USA) per sample and dried with nitrogen. Cell pellets were sonicated in 200 μL PBS, and the resulting lysates were transferred to glass tubes with 1.5 mL of UPLC grade methanol. An aliquot of the lysate was used for protein quantification, using a BCA protein assay kit. Lipid extractions were performed using 5 mL of meth-tert-butyl ether (MTBE) [34] with continuous shaking for 60 min at room temperature (RT). Thereafter, 1.2 mL ddH_2_O was added, and samples were mixed and spun at 1,000 g for 10 min at RT to establish phase separation. The upper organic phase was collected. The remaining aqueous phase was re-extracted with 5 mL MTBE, 1.5 mL methanol, and 1.2 mL ddH_2_O, and the organic phase was collected. The resulting organic phases were dried under a stream of nitrogen, and lipids were reconstituted in 1:1 (v/v) CHCl_3_:MeOH. The extract was re-suspended and diluted 20 times using 2:1:1 (v/v/v) isopropanol:acetonitrile:ddH_2_O for UPLC-MS ESI+ analysis.

Chromatographic separation was modified from [35] using an AQUITY-UPLC system (Waters Corporation, Milford, MA, USA) equipped with a Waters CSH (2.1 × 100 mm, 1.7 μm; CSH pre-column) starting with a 20 minute separation with a linear gradient at 60% solvent A (ddH_2_O:acetonitrile, 40/60, v/v, 10 mM ammonium formate and 0.1% formic acid) and 40% solvent B (actetonitrile:isopropanol, 10/90, v/v, 10 mM ammonium formate and 0.1% formic acid).

A XEVO TQSµ Tandem-Mass Spectrometer equipped with an electrospray ionization source was used for detection. Lipid species were analyzed by multiple reaction monitoring (DG: [MNH4]+ to [RCOO+58]+ of the respective esterified fatty acid, Cone Voltage (CV): 26 V, Collision Energy (CE): 20 V, 58 ms; TG: [MNH4]+ to [DG-H_2_O]+ of the respective DG, CV: 46 V, CE: 30, 67 ms). Lipid species/groups were analyzed with TargetLynx XS Software (Waters, Milford, MA, USA). Data were normalized for recovery, extraction, and ionization efficacy by calculating analyte/ISTD ratios (AU) and expressed as AU/mg protein.

### 2.7. Lipolysis Assay

Differentiated adipocytes (day 8) were washed twice in DMEM + 5 mM glucose and incubated with DMEM containing 2% fatty acid free BSA (FAF-BSA) and 5 mM triacsin C (hereby referred to as base media) supplemented with 2.5 mg/mL WPI and WPH for 4 h. Basal lipolysis was induced by incubating adipocytes in 1 mL base media for 1 h, following which a media aliquot was collected for analysis. Adipocytes were washed once, and lipolysis was stimulated by incubating adipocytes in 1 mL of base media containing 20 µM isoproterenol for 1 h. For experiments involving insulin, either 0 or 100 nM insulin was added during both basal and stimulated lipolysis. After collection of a media aliquot, cells were washed once, harvested, and stored at -80 °C along with media aliquots until further use. Levels of non-esterified fatty acids (NEFA) in the media were determined using a colorimetric kit assay (HRSeries NEFA-HR (2) kit from Wako Chemicals, Richmond, VA, USA) as per the manufacturer’s instructions. Cell pellets were sonicated to obtain protein lysates, and lysate protein concentrations were used to normalize the NEFA levels.

### 2.8. Mitochondrial Analysis

Respiratory oxygen flux in 3T3-L1 adipocytes and C2C12 myotubes was measured in high-resolution using the Oxygraph-2k (OROBOROS Instruments, Innsbruck, Austria) as described [32]. Briefly, following incubation with whey proteins and peptides, cells were detached, resuspended at a concentration of 200,000 cells/mL, and permeabilized with 3 µg/mL digitonin. The respirometry protocol involved the sequential addition of substrates, inhibitors and titration of substrates, as follows: 0.5 mM malate (M), 50 µM palmitoylcarnitine (PC), 5 mM ADP, 0.5 µM carbonyl cyanide-4-(trifluoromethoxy)phenylhydrazone (FCCP). Thereafter, cells were collected and lysed, and lysate protein concentration determined using a BCA protein assay. Mitochondrial respiration was normalized to the protein content.

### 2.9. Citrate Synthase Activity Assay

Citrate synthase activity was determined as previously described, with minor modifications [36]. 3T3-L1 adipocytes and C2C12 myotubes were sonicated in buffer containing 20 mM HEPES, 10 mM EDTA, and 10 µL/mL protease inhibitor, pH 7.4, and incubated on ice for 30 min. Samples were spun at 600 g for 20 min at 4 °C. An aliquot of the supernatant was taken to measure protein concentrations for the purpose of normalizing citrate synthase activity. The resulting supernatant was flash-frozen in liquid nitrogen and stored at −80 °C for 1 h to liberate citrate synthase from the mitochondrial matrix. The reaction was initiated by the addition of 227.5 µL reaction buffer containing 20 mM HEPES, 2 mM EGTA, 220 mM sucrose, 40 mM KCl, 0.1 mM 5,5′-Dithiobis(2-nitrobenzoic acid) (DTNB), 0.3 mM acetyl-CoA, pH 7.4 at 25 °C. After 5 min, a baseline reading was obtained at 412 nm. Thereafter, 0.5 mM oxaloacetate was added, and the increase in absorbance at 412 nm was monitored for 10 minutes.

### 2.10. Statistical Analysis

Results are expressed as mean ± standard error of the mean (SEM). Comparisons between two groups were performed using an unpaired two-tailed Student’s t-test. Comparisons between multiple groups were performed using an unpaired one- or two- way analysis of variance (ANOVA) followed by a Tukey or Sidak post hoc test, as appropriate. All statistical analysis was performed using Prism (GraphPad Software, San Diego, CA, USA). *p*-values of less than 0.05 were considered statistically significant.

## 3. Results

### 3.1. Profile of Peptides in Whey Protein Hydrolysate

Using the bovine proteome FASTA from UniProtKB, shotgun peptidome analysis resulted in the identification of 263 whey peptides in WPH. These peptides were derived from 18 milk protein groups; see  Supplementary File S1 for protein list, peptide sequences, and other information. The chain length and molecular weight of the peptides ranged from 8 to 25 and 0.87 to 3 kDa, respectively. The number of peptides distributed across the different molecular weight ranges are as follows: <1 kDa (9), 1–1.5 kDa (85), 1.5–2 kDa (112), 2–2.5 kDa (44), and 2.5–3 kDa (13). The sequence coverages of 84.8%, 79.7%, and 25.2% were observed for major whey proteins, β-lactoglobulin, α-lactalbumin, and serum albumin (BSA), respectively. Consequently, as shown in Table 2, β-lactoglobulin and α-lactalbumin jointly contributed the majority (~70%) of the identified whey peptides (razor + unique) while BSA contributed 6% of the peptides. Other notable peptide contributors include β-casein and κ-casein, which are often present as residues in whey protein products. The peptidomics approach used in this study is the most comprehensive for identifying peptides in complex mixtures and food matrices. Despite the strength of this approach, it is still challenging to identify peptides accurately with molecular weights smaller than 0.8 kDa [37].

### 3.2. Whey Peptides Promote Differentiation of 3T3-L1 Adipocytes

**Figure 1:**
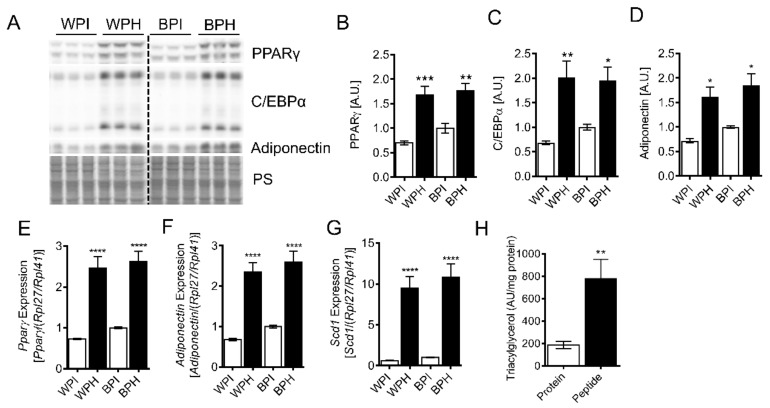
Effect of whey peptides on 3T3-L1 adipocyte differentiation. 3T3-L1 preadipocytes were differentiated in the presence of 2.5 mg/mL WPI, WPH, BPI or BPH. Immunoblotting and densitometric analysis of (**A**,**B)** PPARγ, (**A**,**C**) C/EBPα, and (**A**,**D**) adiponectin (*n* = 6). mRNA levels of PPARγ (**E**) and PPARγ target genes, (**F**) adiponectin and (**G**) stearoyl-CoA desaturase (Scd1) (*n* = 8) were determined. TG levels (**H**) were measured using a targeted lipidomics approach in adipocytes incubated with BSA protein isolate or whey peptides (*n* = 8). (**B**–**H**): * *p* < 0.05, ** *p* < 0.01, *** *p* < 0.001, **** *p* < 0.0001 vs. protein isolate controls. BPI, BSA protein isolate; BPH, BSA peptide hydrolysate; WPI, whey protein isolate; WPH, whey peptide hydrolysate; PPARγ, peroxisome proliferator-activated receptor γ; C/EBPα, CCAAT/enhancer-binding protein α; PS, protein stain; Scd1, stearoyl-Coenzyme A desaturase 1; A.U., arbitrary units.

To determine whether a whey-derived peptide mixture can influence adipocyte differentiation, we incubated 3T3-L1 preadipocytes with peptide-containing whey protein hydrolysate (WPH) or unhydrolyzed whey protein isolate (WPI, control) during the differentiation process. After the 8-day differentiation protocol, 3T3-L1 adipocytes incubated with WPH had increased mRNA and protein levels of peroxisome proliferation activator receptor γ (PPARγ) and CCAAT/enhancer-binding protein α (C/EBPα), primary regulators of adipocyte differentiation, compared to the adipocytes differentiated in presence of WPI (Figure 1A–C,E). In agreement with the upregulation of PPARγ, the PPARγ targets, adiponectin, and stearoyl-CoA desaturase (SCD1) were also increased in adipocytes differentiated in the presence of WPH (Figure 1A,D–G). BSA is a significant component of whey, yet it remains relatively unexplored whether BSA-derived peptides have metabolic effects. To determine whether peptides derived from BSA could contribute to the differentiation-stimulating effect of WPH, we co-incubated 3T3-L1 cells with BSA peptides (BPH) or native protein (BPI). BPH but not BPI resulted in similar upregulation of adipocyte differentiation markers and PPARγ targets compared to WPH (Figure 1A–G). Whey peptide-induced increases in differentiation markers corresponded with elevated adipocyte TG levels (Figure 1H). Taken together, these results suggest that whey peptides promote adipocyte differentiation and lipid accumulation, an effect that is at least partially mediated by peptides derived from BSA.

### 3.3. Whey Peptides Increase Lipolysis in 3T3-L1 Adipocytes

**Figure 2:**
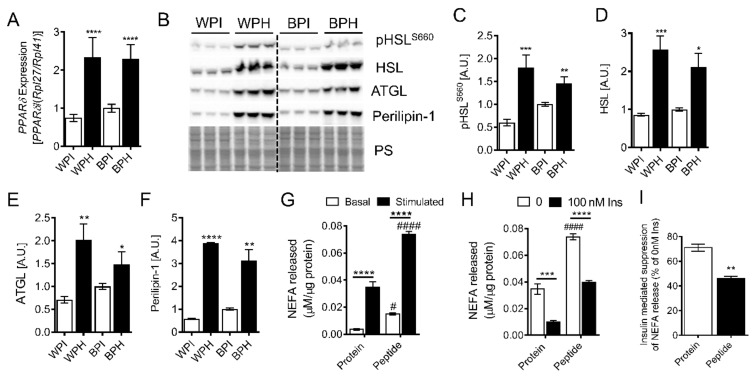
Influence of whey peptides on lipolysis in 3T3-L1 adipocytes. 3T3-L1 preadipocytes were differentiated in presence of 2.5 mg/mL WPI, WPH, BPI, or BPH. (**A**) *PPARδ* mRNA levels were determined (*n* = 8). Immunoblotting and densitometric analysis were performed to assess protein levels of (**B**,**C**) pHSL^S660^, (**B**,**D**) HSL, (**B**,**E**) ATGL, and (**B**,**F**) perilipin-1 (*n* = 6). (**G**) Basal and isoproterenol-stimulated lipolysis and (**H**,**I**) insulin-mediated suppression of lipolysis was determined in adipocytes incubated with BSA protein isolate or whey peptides (*n* = 6). **A**, **C**–**I**: * *p* < 0.05, ** *p* < 0.01, *** *p* < 0.001, **** *p* < 0.0001 vs. protein isolate controls or as indicated. ^#^ *p* < 0.05, ^####^ *p* < 0.0001 in peptide vs. protein-treated adipocytes. HSL, hormone-sensitive lipase; ATGL, adipose triglyceride lipase; PS, protein stain; NEFA, non-esterified fatty acids; A.U., arbitrary units.

To examine whether whey peptides influence adipocyte lipolysis, we determined the expression of proteins involved in lipolysis and lipid metabolism in 3T3-L1 adipocytes differentiated in the presence of WPH, BPH and native proteins (WPI, BPI). mRNA levels of PPARδ, a master regulator of lipolysis and fat oxidation, were increased more than two-fold in adipocytes incubated with peptides (BPH, WPH) vs. native proteins (BPI, WPI) (Figure 2A). In agreement with increased PPARδ levels, protein levels of hormone-sensitive lipase (HSL) phosphorylated at Ser660, total HSL and adipose triglyceride lipase (ATGL) were significantly increased by WPH and BPH treatment (Figure 2B–E). Protein levels of perilipin-1, a lipid droplet coat protein that regulates lipolysis via interaction with HSL and the ATGL co-activating protein, CGI58, in a protein kinase A-dependent manner, was also increased by peptide co-incubation (Figure 2B,F). The upregulation of proteins involved in lipolysis was associated with increased basal and isoproterenol-stimulated NEFA release from adipocytes incubated with WPH compared to native protein (Figure 2G). To determine whether adipocytes incubated with whey peptides had altered insulin sensitivity, we measured NEFA release in the presence and absence of insulin. As expected, insulin markedly reduced isoproterenol-stimulated lipolysis (Figure 2H). However, insulin-mediated suppression of lipolysis was less pronounced in adipocytes incubated with WPH compared to native protein control (Figure 2I). Taken together, these results suggest that whey-derived peptides increase lipolytic activity in adipocytes.

### 3.4. Whey Peptides Enhance Mitochondrial Fatty Acid Oxidation in 3T3-L1 Adipocytes

**Figure 3:**
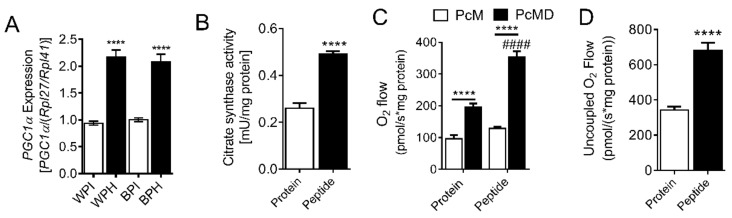
Effect of whey peptides on mitochondrial function in adipocytes. 3T3-L1 preadipocytes were differentiated in the presence of 2.5 mg/mL WPI, WPH, BPI, or BPH. (**A**) *Pgc1α* mRNA levels and (**B**) citrate synthase activity (*n* = 7–8). (**C**) Fatty acid-linked mitochondrial respiration and (**D**) uncoupled respiration was assessed in permeabilized adipocytes incubated with BSA protein isolate or whey peptides (*n* = 7). (**A**–**D**): **** *p* < 0.0001 vs. protein controls or as indicated. ^####^ *p* < 0.0001 for peptide vs. protein-treated adipocytes. Pgc1α, PPARγ coactivator 1-α; Pc, palmitoylcarnitine; M, malate; D, ADP.

In addition to increasing the expression of proteins involved in lipolysis, PPARδ also activates mitochondrial fat oxidation. Since PPARδ was upregulated by WPH and BPH, we examined the influence of these peptides on adipocyte mitochondria. Levels of PGC1α, a master regulator of mitochondrial biogenesis, was increased in adipocytes differentiated in the presence of WPH and BPH compared to native proteins (Figure 3A). Similarly, citrate synthase activity, an index of mitochondrial content [38], was also increased by whey peptides (Figure 3B). We employed high-resolution respirometry in permeabilized adipocytes to determine whether mitochondrial respiration was altered by peptide treatment. Fatty-acid linked respiration was increased two-fold in adipocytes incubated with whey peptides vs. native protein (Figure 3C). Furthermore, maximal respiration in the presence of FCCP, a mitochondrial uncoupler, was also upregulated by peptide co-treatment (Figure 3D). Taken together, these data suggest that whey peptides increase mitochondrial content and fat oxidation in adipocytes.

### 3.5. Whey Peptides Ameliorate Palmitate-Induced Insulin Resistance in C2C12 Myotubes

**Figure 4:**
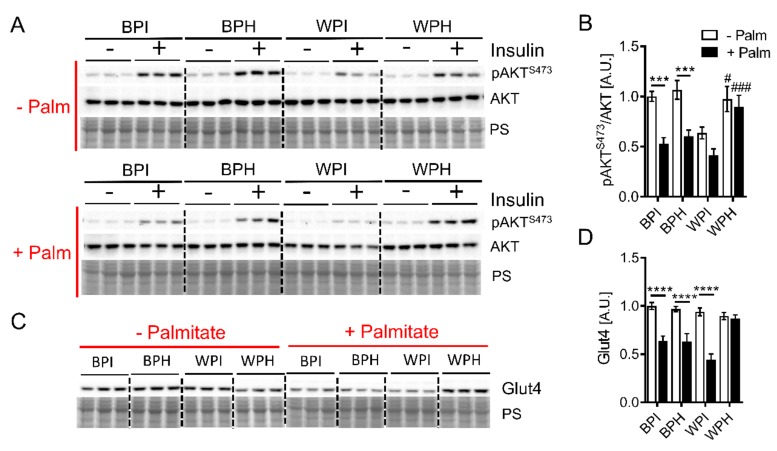
Effect of whey peptides on insulin signaling in C2C12 myotubes. C2C12 myotubes were incubated in the presence or absence of 0.4 mM palmitate and co-incubated with 2.5 mg/mL BPI, BPH, WPI, or WPH. Immunoblotting and densiometric analysis were performed to assess (**A**,**B**) AKT phosphorylation at S473 and (**C**,**D**) Glut4 protein levels (*n* = 6). (**B**,**D**): *** *p* < 0.001, **** *p* < 0.0001 as indicated. ^#^ *p* < 0.05, ^###^ *p* < 0.001 for peptide vs. protein treated myotubes. A.U., arbitrary units.

Skeletal muscle insulin resistance is a hallmark of metabolic syndrome and is influenced by changes in adipose metabolism. Therefore, we next aimed to determine whether dietary whey peptides influence insulin sensitivity and metabolism in skeletal muscle cells. C2C12 myotubes were incubated for 18 h in the presence of whey and BSA peptides or their native (unhydrolyzed) protein controls. Concurrently, cells were incubated in the presence or absence of palmitate, which triggers lipotoxicity and insulin resistance [32]. In the absence of palmitate, insulin-stimulated phosphorylation of AKT at S473 was similar between myotubes incubated with WPH, BPI, and BPH, as evidenced by a 4- to 5-fold increase in AKT phosphorylation relative to no-insulin controls (Figure 4A). Interestingly, WPI treatment tended to lower insulin-stimulated AKT phosphorylation compared to other groups; incubation with insulin resulted in only a ~two-fold increase in AKT phosphorylation compared to the no-insulin control (Figure 4A). Palmitate induced insulin resistance in C2C12 myotubes, as evidenced by a two-fold reduction in the insulin-stimulated AKT ^S473^ phosphorylation in BPI and BPH groups (Figure 4A,B). Insulin-stimulated AKT phosphorylation in palmitate-treated cells co-incubated with WPI was similar compared to cells co-incubated with BPI and BPH (Figure 4A,B). However, co-incubation with WPH prevented a palmitate-mediated decline in insulin-stimulated AKT phosphorylation and maintained a four-fold increase in AKT phosphorylation in response to insulin relative to the no-insulin control (Figure 4A,B). Corresponding with preserved insulin signaling in cells co-incubated with palmitate and WPH, protein levels of Glut4, the major insulin-sensitive glucose transporter in skeletal muscle, were maintained in this group while palmitate treatment led to decreased Glut4 levels in BPI, BPH, and WPH groups (Figure 4C,D). Taken together, these results suggest that whey peptides other than those derived from BSA ameliorate palmitate-induced insulin resistance in C2C12 myotubes.

### 3.6. Whey Peptides Protect from Palmitate-Induced Inflammation and Endoplasmic Reticulum (ER) Stress, Which is Associated with Decreased DG Accumulation in C2C12 Myotubes

**Figure 5:**
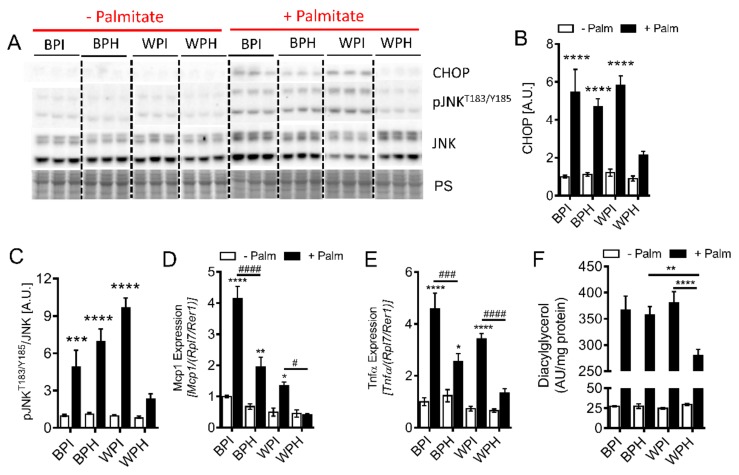
Influence of whey peptides on palmitate-induced ER stress, inflammation, and DG accumulation in C2C12 myotubes. C2C12 myotubes were incubated in the presence or absence of 0.4 mM palmitate and co-incubated with 2.5 mg/mL BPI, BPH, WPI or WPH. Immunoblotting and densiometric analysis were performed to assess (**A**,**B**) protein levels of CHOP and (**A**,**C**) phosphorylation JNK at T183/Y185 (*n* = 6). mRNA levels of inflammatory markers, (**D**) *Mcp1* and (**E**) *Tnfα* (*n* = 8) were determined. Levels of (**F**) DGs were measured using a targeted lipidomic approach (*n* = 6–8). (**B**–**E**): * *p* < 0.05, ** *p* < 0.01, *** *p* < 0.001, **** *p* < 0.0001 vs. no palmitate controls or as indicated. ^#^ *p* < 0.05, ^###^ *p* < 0.001, ^####^ *p* < 0.0001 as indicated. CHOP, C/EBP homologous protein; JNK, c-Jun N-terminal kinase; *Mcp1*, monocyte chemoattractant protein 1; *Tnfα*, tumor necrosis factor α; Palm, palmitate; A.U., arbitrary units.

Palmitate is an inducer of ER stress and inflammation, both of which are implicated in insulin resistance [39,40]. Therefore, we examined whether whey peptides alter markers of palmitate-induced ER stress and inflammation. Palmitate incubation led to increased protein levels of C/EBP homologous protein (CHOP), a marker of ER stress, in all groups except in cells incubated with WPH (Figure 5A,B). Similarly, palmitate increased c-Jun N-terminal kinase (JNK) phosphorylation at T183/Y185 and mRNA levels of *Mcp1* and *Tnfα,* severalfold with BPI, BPH, and WPI co-incubation, while palmitate failed to upregulate these inflammatory markers upon co-incubation with WPH (Figure 5A,C–E). Palmitate may stimulate insulin resistance and inflammation via increased cellular accumulation of toxic lipid species, including DGs. Therefore, we determined whether whey peptides affect DG accumulation. While palmitate incubation led to increased DG levels in all groups, DG accumulation was reduced by 30% in myotubes co-incubated with WPH compared to WPI and BPH groups (Figure 5F). Taken together, these results suggest that amelioration of palmitate-induced insulin resistance in myotubes by whey peptides is mediated by reduced ER stress, inflammation, and DG accumulation.

### 3.7. Whey Peptides Increase TG Accumulation in C2C12 Myotubes

**Figure 6:**
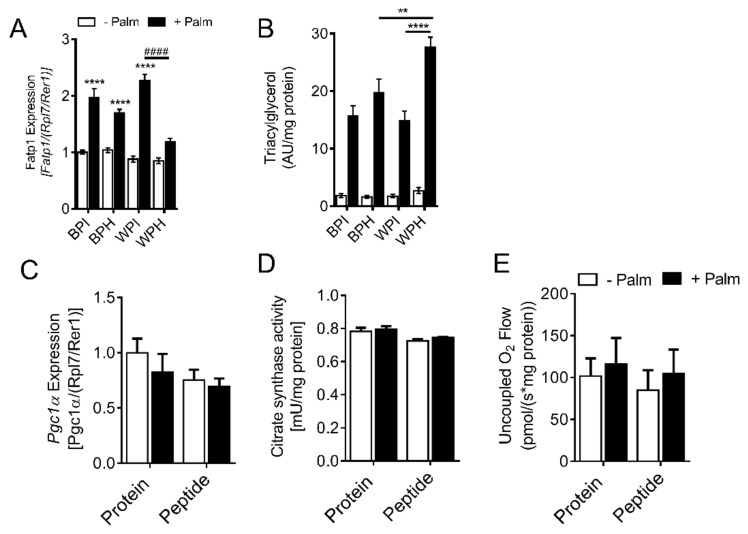
Effect of whey peptides on fatty acid transporter levels, TG accumulation, and mitochondrial abundance. C2C12 myotubes were incubated in the presence or absence of 0.4 mM palmitate and co-incubated with 2.5 mg/mL BPI, BPH, WPI, or WPH. (**A**) mRNA levels of *Fatp1* (*n* = 8) were assessed. (**B**) TG levels were measured using a targeted lipidomics approach (*n* = 8). (**C**) *Pgc1α* mRNA levels (*n* = 8), (**D**) citrate synthase activity (*n* = 3), and (**E**) uncoupled respiration (*n* = 7) were determined in myotubes treated with BSA protein isolate or whey peptides. ** *p* < 0.01, **** *p* < 0.0001 vs. no palmitate controls or as indicated, ^####^ *p* < 0.0001 as indicated. *Fatp1*, fatty acid transport protein 1; *Pgc1α*, peroxisome proliferator-activated receptor γ coactivator 1-α; A.U., arbitrary units.

Reduced DG accumulation in palmitate-treated myotubes co-incubated with WPH could result from decreased fatty acid import, increased incorporation of fatty acids into the TG pool, and increased mitochondrial fatty acid oxidation. To address this, we measured mRNA levels of the fatty acid transporter, FATP1 (Figure 6A). While palmitate treatment increased FATP1 levels in the presence of BPI, BPH, and WPI, palmitate did not affect FATP1 levels in myotubes co-incubated with WPH (Figure 6A), suggesting that WPH may reduce palmitate uptake via FATP1 into myotubes. Interestingly, palmitate incubation resulted in significantly increased TG accumulation in WPH-treated myotubes compared to BPH and WPI groups (Figure 6B), corresponding with decreased DG levels (Figure 5E), suggesting that palmitate is increasingly shunted towards TG synthesis in the WPH group. To examine whether whey peptide incubation influences mitochondrial content and respiration in C2C12 myotubes, we examined mRNA levels of *Pgc1α* and citrate synthase activity. Unlike adipocytes, peptide treatments had no effect on these indices of mitochondrial biogenesis/content in C2C12 myotubes, when compared to the native protein controls (Figure 6C,D). Similarly, uncoupled respiration in permeabilized C2C12 myotubes was unchanged by peptide treatment (Figure 6E). Taken together, these data suggest that whey peptides, other than those derived from BSA, reduce fatty acid transport via FATP1 and increase TG accumulation in myotubes exposed to palmitate without influencing mitochondrial abundance and function.

## 4. Discussion

Insulin resistance in adipose tissue and skeletal muscle drives the development of systemic insulin resistance, which can progress to type 2 diabetes [41]. Insulin sensitization of these tissues, therefore, can potentially reduce and prevent systemic insulin resistance and its complications. Recent studies have demonstrated that hydrolysis of milk proteins, including whey proteins, generates bioactive peptides that can affect glucose homeostasis [18,24,29]. However, it remained unclear whether and how whey peptide exposure affects lipid metabolism and insulin sensitivity in adipose tissue and skeletal muscle.

In this study, we demonstrate that hydrolyzed whey protein promotes adipocyte differentiation and TG storage, corresponding with PPARγ activation, and increases mitochondrial abundance and fat oxidation. Our data also suggest that these lipid metabolism-stimulating effects of whey peptides are at least in part mediated by peptides derived from BSA, a major component of whey protein. Interestingly, the 16 BSA-derived peptides identified in WPH (with chain length and molecular weight ranges of 8–22 and 1.0–2.4 kDa, respectively; Supplementary Information) have not been previously reported as bioactive. Previous studies showed mixed effects of milk peptides on PPARγ activation. Peptides from casein glycomacropeptides or tuna fish decreased PPARγ protein levels and lipid accumulation in primary rat and 3T3-L1 preadipocytes, respectively [42,43]. Conversely, Chakrabarti and Wu [18] showed that synthetic lactotripeptides, IPP and VPP, originally resulting from casein digestion during fermentation, upregulate PPARγ levels and lipid storage in 3T3-F442A cells. Our data suggest that a bioactive peptide mixture from whey protein digested with pepsin and pancreatin has an overall activating effect on PPARγ, which is supported by increased PPARγ protein levels, upregulation of PPARγ-sensitive genes, and increased TG accumulation. Interestingly, adipocyte PPARδ levels were also augmented with whey peptides. To our knowledge, this is the first study to demonstrate that bioactive peptides can increase PPARδ, a ubiquitously expressed transcription factor that serves as a master regulator of lipolysis and lipid oxidation [44]. Consistent with increased PPARδ, protein levels of lipolytic enzymes, basal and isoproterenol stimulated lipolysis, and fatty acid-linked mitochondrial respiration was elevated following whey peptide incubation. The concurrent activation of both PPARγ and PPARδ was unexpected, as these proteins typically have opposing roles by promoting lipid storage and oxidation, respectively [44,45], although PPARδ may enhance PPARγ-stimulated adipocyte differentiation [46]; it is conceivable that distinct whey peptides within the hydrolysate mixture mediate PPARγ and PPARδ upregulation. The identity of these particular groups of peptides is currently unknown, and further research is required to identify potential PPARγ and PPARδ activators in pepsin-pancreatin-digested whey protein and BSA. Notably, PPARδ agonists are developed for the improved treatment of metabolic syndrome [47]. Thus, whey and, specifically, BSA derived peptides could serve as a valuable reservoir of novel PPARδ regulators and agonists.

Interestingly, prior studies demonstrated that whey protein supplementation has fat mass, reducing effects in overweight and obese humans [48,49]. Similarly, high fat-fed mice consuming whey proteins were protected from early life weight gain and displayed improved glucose tolerance [50], suggesting that whey protein consumption has an anti-adipogenic effect. In our study, pre-digestion of whey and BSA proteins into peptides had a marked adipogenic effect on cultured preadipocytes compared to native unhydrolyzed protein controls. In future studies employing dietary whey protein supplementation, it would be valuable to determine which proportion of consumed whey protein is converted to bioactive peptides in the gut and reaches the circulation in mice and humans as it appears that whey protein and peptides may have different effects on adipogenesis.

Few prior studies have examined the effect of dietary peptides on skeletal muscle insulin sensitivity. Soybean-derived peptides improved glucose transport and insulin signaling in high fat diet-fed mice and in insulin-resistant C2C12 myotubes via upregulation of insulin receptor and insulin receptor substrate 1 and activation of AMPK [51,52]. Whey peptides were also previously shown to stimulate glucose uptake, glycogen synthesis, and AMPK activity [24,27,29]. Our current work complements these studies by showing that whey peptide treatment ameliorates lipotoxicity-induced insulin-resistance in C2C12 myotubes. Interestingly, unlike adipocytes, the bioactive whey peptides responsible for improving insulin sensitivity in myotubes do not appear to originate from BSA. Future work will be required to determine which protein component of whey and specific bioactive peptides are responsible for the effects of whey peptide mixture on insulin sensitivity in myotubes.

In our study, whey peptides had a potent anti-inflammatory effect by reducing JNK phosphorylation and mRNA levels of *Tnfα* and *Mcp1*, similar to the anti-inflammatory effects of tripeptides from casein [18] and soy protein [53]. To our knowledge, our study is the first to implicate reduced levels of DG species in whey peptide-mediated protection from lipotoxicity-induced insulin resistance in myotubes. As expected, palmitate incubation raised DG levels ~15-fold in BPI, BPH, and WPI treated myotubes, while palmitate incubation also resulted in increased DG levels in myotubes incubated with WPH, DG levels remained lower in the WPH group compared to other groups. Increased DG accumulation in skeletal muscle is evidenced in high fat-fed rodents and humans with diabetes [54]. DGs promote insulin resistance in skeletal muscle by recruiting protein kinase C isoforms to the plasma membrane, which phosphorylate and inactivate the insulin receptor [54]. Although DG levels serve as a prominent marker for lipotoxicity, palmitate is also known to increase levels of other potentially toxic lipid species, including ceramides [14]. It is possible that whey peptides also influence ceramide content in myotubes, which should be assessed in future studies.

Whey peptide incubation appears to alter the metabolic fate of palmitate in muscle cells. Based on our data, we propose that whey peptides may reduce DG accumulation in the presence of palmitate through two mechanisms. The first involves the import of fatty acids; WPH co-incubation reduced levels of FATP1, a major fatty acid transporter in skeletal muscle, which may limit palmitate uptake. Secondly, WPH co-incubation resulted in increased TG accumulation, suggesting that palmitate is increasingly shunted towards TG synthesis and sequestered in the lipid droplet. Prior studies have established that the TG pool per se does not stimulate insulin resistance in skeletal muscle and that sequestration of fatty acids in the TG pool is rather beneficial, as it prevents fatty acid-mediated apoptotic pathway activation [55,56]. This notion is also supported by the fact that muscle TG levels are elevated in highly insulin sensitive endurance-trained athletes [57]. The effect of WPH on myotube lipid accumulation does not appear to be due to changes in mitochondrial abundance or fat oxidation.

Taken together, our study suggests that whey peptides generated via pepsin-pancreatin digestion have pleiotropic metabolic effects on adipocytes and skeletal myotubes. Whey peptides, specifically those derived from BSA, promote adipocyte differentiation and concurrent activation of PPARγ and PPARδ to increase lipid storage and oxidation, respectively. In myotubes, whey peptides ameliorate lipotoxicity-induced inflammation, ER stress, and DG accumulation, and increase sequestration of fatty acids in the TG pool, thereby countering insulin resistance. These data provide a rationale for determining the effect of whey peptide consumption on adipose and muscle lipid metabolism and insulin function in preclinical models *in vivo*.

## Figures and Tables

**Table 1:** Primers used in this study.

Target	Primer Sequence (5’ to 3’)
*Pparγ* F	ACATAAAGTCCTTCCCGCTGA
*Pparγ* R	TCGAAACTGGCACCCTTGAAAA
*Adipoq* F	AGCCGCTTATGTGTATCGC
*Adipoq* R	GTCCCGGAATGTTGCAGTAGAAC
*Scd1* F	TTACGACCGGAAGAAAGTT
*Scd1* R	ATTAACACCCCGATAGCAATA
*Pparδ* F	TCATTGAGCCCAAGTTCGAGT
*Pparδ* R	CCGGTCTCCACACAAAATGAT
*Pgc1α* F	TTTGCCCAGATCTTCCTGAAC
*Pgc1α* R	TCGCTACACCACTTCAATCCA
*Mcp1* F	TCGGAACCAAATGAGATCAGA
*Mcp1* R	CAGATTTACGGGTCAACTTC
*Tnfα* F	CATCCATTCTCTACCCAGCCC
*Tnfα* R	CATGAGAGGCCCACAGTCCA
*Fatp1* F	CCTCTGGGCACCATTCTATATTC
*Fatp1* R	ACACTAGCCACATCCAAGTGA
*Rpl27* F	ACGGTGGAGCCTTATGTGAC
*Rpl27* R	TCCGTCAGAGGGACTGTCTT
*Rpl41* F	GCCATGAGAGCGAAGTGG
*Rpl41* R	CTCCTGCAGGCGTCGTAG
*Rpl7* F	ACGGTGGAGCCTTATGTGAC
*Rpl7* R	TCCGTCAGAGGGACTGTCTT
*Rer1* F	GCCTTGGGAATTTACCACCT
*Rer1* R	CTTCGAATGAAGGGACGAAA

**Table 2:** Protein groups and the number of peptides identified in the whey protein hydrolysate generated with pepsin-pancreatin.

ID	Whey Protein Groups Identified	Major Protein UniProtKB Accession Number	Number of Peptides Identified	% of Total Number of Peptides
1	Alpha-lactalbumin	P00711	69	26.2
2	Alpha-S2-casein	P02663	1	0.4
3	Beta-casein	P02666	21	8.0
4	Kappa-casein	P02668	17	6.5
5	Serum albumin (BSA)	P02769	16	6.1
6	Osteopontin	P31096	1	0.4
7	Serotransferrin	Q29443	2	0.8
8	Ig-like domain-containing protein	F1MLW7	2	0.8
9	Alpha-amylase	Q3MHH8	2	0.8
10	Folate receptor alpha	P02702	2	0.8
11	Beta-lactoglobulin	P02754	115	43.7
12	Lactotransferrin	P24627	2	0.8
13	NPC intracellular cholesterol transporter 2	P79345	4	1.5
14	Glycosylation-dependent cell adhesion molecule 1	P80195	2	0.8
15	Lactadherin	G3MYW7	1	0.4
16	Sortilin related VPS10 domain containing receptor 1	A0A3Q1LSH9	1	0.4
17	Multiple coagulation factor deficiency 2	Q3MHJ4	1	0.4
18	Uncharacterized protein	A0A3Q1M3L6	4	1.5

## Supplementary Materials

The following are available online at https://www.mdpi.com/2072-6643/12/2/425/s1, File S1. List of whey protein groups and whey and BSA peptides.
